# HNF4G increases cisplatin resistance in lung adenocarcinoma via the MAPK6/Akt pathway

**DOI:** 10.7717/peerj.14996

**Published:** 2023-03-10

**Authors:** Jiaqi Liang, Guangyin Zhao, Yunyi Bian, Guoshu Bi, Qihai Sui, Huan Zhang, Haochun Shi, Guangyao Shan, Yiwei Huang, Zhencong Chen, Lin Wang, Cheng Zhan

**Affiliations:** Department of Thoracic Surgery, Zhongshan Hospital, Fudan University, Shanghai, China

**Keywords:** Lung adenocarcinoma, Cisplatin, HNF4G, MAPK6, Transcription factor

## Abstract

**Background:**

Lung adenocarcinoma is one of the most common tumors, and cisplatin is frequently used in treating lung adenocarcinoma patients. This study aimed to look into the roles and mechanisms of HNF4G in cisplatin resistance of lung adenocarcinoma.

**Materials & Methods:**

Cisplatin resistance and gene expression data of 542 cell lines from the CTRP and CCLE databases were analyzed. HNF4G expression was detected in the lung adenocarcinoma cell lines after treatment with various concentrations of cisplatin. Cisplatin sensitivity curves were detected in cells that overexpressed or knocked down HNF4G. The ChIP-Seq data were then analyzed to identify the targets of HNF4G involved in cisplatin resistance. Expression and phosphorylation of the MAPK6/Akt pathway were detected after HNF4G was overexpressed or knocked down. Finally, ChIP-qPCR and dual-luciferase assays were used to investigate the regulation of HNF4G on MAPK6.

**Results:**

In cell lines, high expression of HNF4G was significantly positively correlated with cisplatin resistance, and lung adenocarcinoma patients who had high HNF4G expression had a poor prognosis. Cisplatin treatment increased HNF4G expression, and overexpression of HNF4G significantly increased the resistance to cisplatin in A549 and HCC827 cells, whereas knockdown of HNF4G had the opposite effect. HNF4G overexpression increased MAPK6 expression and activated the MAPK6/Akt pathway, while an Akt inhibitor reduced the effects of HNF4G on cisplatin resistance. HNF4G bound to the MAPK6 promoter region, promoting MAPK6 expression, according to ChIP-qPCR and luciferase assays.

**Conclusion:**

By binding to the MAPK6 promoter region, HNF4G promotes MAPK6 expression and subsequent Akt phosphorylation, resulting in resistance to cisplatin in lung adenocarcinoma.

## Introduction

In recent years, lung cancer has become the second most frequently diagnosed cancer and the leading cause of tumor death (Sung et al., 2021). The most common pathological subtype of lung cancer is lung adenocarcinoma, which accounts for nearly two-thirds of all new lung cancer cases yearly (Lu et al., 2019). However, due to the strong proliferation ability and high malignancy of lung adenocarcinoma, the treatment effect and prognosis of lung adenocarcinoma patients are still not ideal at this time. The overall 5-year survival rate is only about 30%, imposing a significant burden on human health, patients’ families, and society (Allemani et al., 2018).

Chemotherapy is still one of the most commonly used treatments for lung adenocarcinoma, especially in patients who have advanced lung adenocarcinoma and are resistant to targeted therapy or immunotherapy (Miller & Hanna, 2021). Understanding the mechanisms of resistance to cisplatin, the most commonly used reagent in chemotherapy, is critical for improving patient outcomes. In this research, we discovered that a transcript factor, hepatocyte nuclear factor 4 gamma (HNF4G), significantly increases cisplatin’s resistance in lung adenocarcinoma by upregulating the MAPK6/Akt signaling pathway. We hope that our findings will aid in the future prognosis of patients.

## Materials & Methods

### Cell culture

The Chinese National Collection of Authenticated Cell Cultures (Shanghai, China) provided two human lung adenocarcinoma cell lines, A549 and HCC827, as well as human embryonic kidney cell line 293T, which were cultured in high-glucose Dulbecco’s modified Eagle’s medium (KeyGEN, Nanjing, Jiangsu, China) with 10% fetal bovine serum (Every Green, Huzhou, Zhejiang, China), 100 U/mL penicillin (Beyotime, Shanghai, China), and 0.1mg/ml streptomycin (Beyotime).

### Lentiviruses and compounds

Lentivirus vectors of HNF4G-Flag, sh-HNF4G-1 (5′-GCACCAGAAGAAGCACATTTG-3′), sh-HNF4G-2 (5′-GCACATTTGATGGCAGCAACA-3′), and corresponding negative control were obtained from GENECHEM (Shanghai, China), transfected into A549, HCC827, and 293T cells, and then screened by puromycin (Beyotime).

Cisplatin and MK-2206 were both obtained from TOPSCIENCE (Shanghai, China).

### Detection of cisplatin sensitivity

To detect the cisplatin sensitivity of cells, 4,000 cells were seeded into 96-well plates and then treated with 0, 1, 2, 4, 8, and 16 uM cisplatin. After 48 h, alamaBlue (YEASEN, Shanghai, China) was added, and the fluorescence was monitored at 545 nm excitation and 590 nm emission wavelengths to detect cell viability.

### Quantitative real-time PCR (qRT-PCR)

As previously described (Liang et al., 2022), total RNA was extracted using TRNzol Universal (TIANGEN, Beijing, China), and cDNA was synthesized using Hifair® III 1st Strand cDNA Synthesis SuperMix for qPCR (YEASEN). Hieff® qPCR SYBR Green Master Mix (Low Rox Plus) (YEASEN) was used for qPCR. The sequences of primers were as follows: HNG4F-F:5′-CAACGGTGTCAACTGTCTGTG-3′, HNG4F-R:5′- AAACGTGACTCTTACGAATGCT-3′ MAPK6-F: 5′-ACGGAAGACTTGGTGCTGAAGAT-3′, MAPK6-R: 5′-TACGCTGAGAAGCTCCTGACGAT-3′, *β*-actin-F: 5′-TGACGTGGACATCCGCAAAG-3′, *β*-actin-R: 5′-CTGGAAGGTGGACAGCGAGG-3′.

### Western blot assay

RIPA Lysis Buffer (Beyotime, Shanghai, China), Protease Inhibitor Cocktail (TOPSCIENCE), and Phosphatase Inhibitor Cocktail III (TOPSCIENCE) were used to extract proteins. SDS-PAGE was used to separate protein samples, which were then transferred to polyvinylidene fluoride membranes. HNF4G (1:3000, PP-N3224-00; R&D System, Minneapolis, MN, US), MAPK6 (1:3000, abs152150; Absin, Shanghai, China), p-MAPK6 (1:1000, AF7407; Affinity, Liyang, Jiangsu, China), Akt (1:3000, CY5561; Abways, Shanghai, China), p-Akt (1:1000, AP0637; Abclonal, Wuhan, China), GAPDH (1:3000, AG019; Beyotime) antibodies were used to detect the specific protein bands.

### Chromatin immunoprecipitation (ChIP) assay

ChIP was performed using SimpleChIP® Plus Enzymatic Chromatin IP Kit (Magnetic Beads) (CST, Boston, MA, US) according to the manufacturer’s instructions. Flag-Tag Antibody (1:100; CST) was used to pull down the HNF4G’s binding chromatin in HNF4G-Flag overexpressing cells. The sequences of primers used in ChIP-qPCR were as follows: primer_1F: 5′-GCTAGGATTACAGGCGTGAACCA-3′, primer_1R: 5′-GCTAGGATTACAGGCGTGAACCA-3′, primer_2F: 5′-ACCATCTTGCTGAGAACCAGACA-3′, primer_2R: 5′- CGGAGGAGCGGATATAACCAGGA-3′, primer_3F: 5′-TGTCGCCTTCCAGCCAATCCA-3′, primer_3R: 5′-CCCAGTGCCAGCGTTTACTAAGC-3′.

### Dual-luciferase reporter assay

The MAPK6 promoter sequences (NM 002748.4) and the corresponding mutated sequences on the predicted target sites of HNF4G were cloned into the firefly luciferase reporter plasmids by GENECHEM. These plasmids were then co-transfected with renilla luciferase reporter plasmids into HNF4G overexpressed or knockdown 293T cells. Dual-luciferase reporter assays were performed 48 h after transfection using the Luciferase Reporter Gene Assay Kit (Beyotime).

### Bioinformatics and statistical analyses

The cisplatin sensitivity and the gene expression data of cell lines were obtained from the Cancer Therapeutics Response Portal (CTRP) website (https://portals.broadinstitute.org/ctrp/) and the Cancer Cell Line Encyclopedia (CCLE) website (https://sites.broadinstitute.org/ccle/) (Rees et al., 2016; Barretina et al., 2012). The correlation between HNF4G and MAPK6 in the lung adenocarcinoma dataset of The Cancer Genome Atlas (TCGA) was obtained from the GEPIA website (http://gepia.cancer-pku.cn/) (Tang et al., 2017). Survival analysis of HNF4G expression in lung adenocarcinoma patients who received chemotherapy was performed using the Kaplan–Meier Plotter website (https://kmplot.com/analysis/) (Gyorffy, 2021). ChIP-Seq data of HNF4G were obtained from the Encode website (https://www.encodeproject.org) (Luo et al., 2020) and the JASPAR website (https://jaspar.genereg.net) (Castro-Mondragon et al., 2022) was used to predict the binding sites of HNF4G.

Student’s *t*-test and ANOVA test were performed to compare variables between groups using R software (version 4.0.3). The *p* values <0.05 were considered significant.

## Results

### High expression of HNF4G is significantly positively correlated with cisplatin resistance in bioinformatic analyses

**Figure 1:**
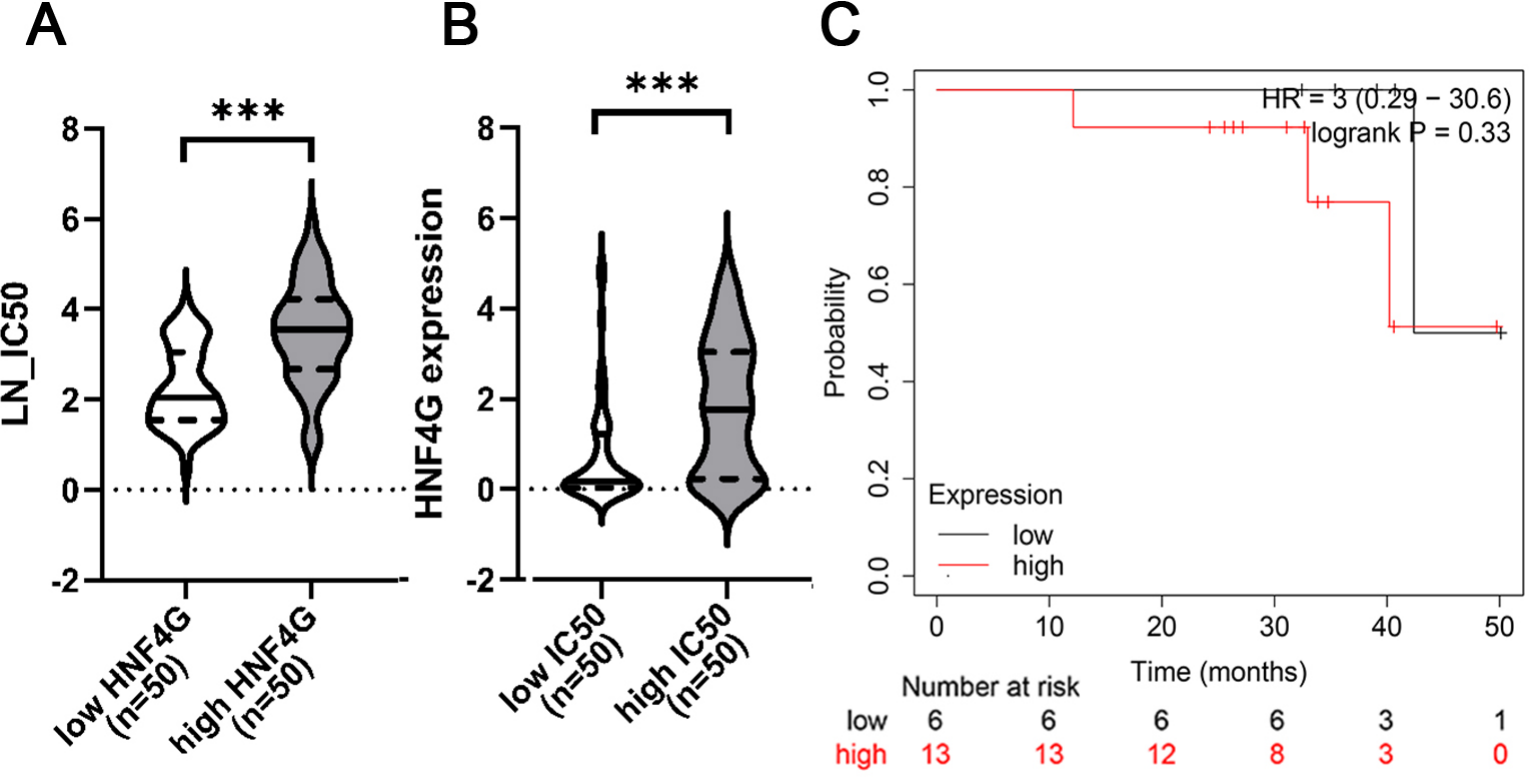
High expression of HNF4G is significantly positively correlated with cisplatin resistance in bioinformatic analyses. (A) The half maximal inhibitory concentrations (IC50s) against cisplatin of the 50 cell lines with the highest HNF4G and the 50 cell lines with the lowest HNF4G expression. (B) The HNF4G expression of the 50 most cisplatin-resistant cell lines and the 50 most cisplatin-sensitive cell lines. (C) The survival curves of lung adenocarcinoma patients with high or low HNF4G expression. *** *p* < 0.001.

When we examined the relationship between cisplatin resistance and gene expression in 542 cell lines from the CTRP and CCLE databases, we discovered that cells with high HNF4G expression were more resistant to cisplatin. As shown in Fig. 1A, the half maximal inhibitory concentrations (IC50s) of the 50 cell lines with the highest HNF4G expression against cisplatin were significantly higher than the IC50s of the 50 cell lines with the lowest HNF4G expression against cisplatin. Simultaneously, HNF4G expression in the 50 most cisplatin-resistant cell lines was significantly higher than in the 50 most cisplatin-sensitive cell lines (Fig. 1B).

Meanwhile, we examined the prognosis of lung adenocarcinoma patients receiving chemotherapy in the Kaplan–Meier Plotter database and discovered that patients with high HNF4G expression had a worse prognosis, despite the fact that the *p*-value was not significant, which may be due to the small sample size (Fig. 1C).

### HNF4G promotes resistance to cisplatin in lung adenocarcinoma cells

**Figure 2:**
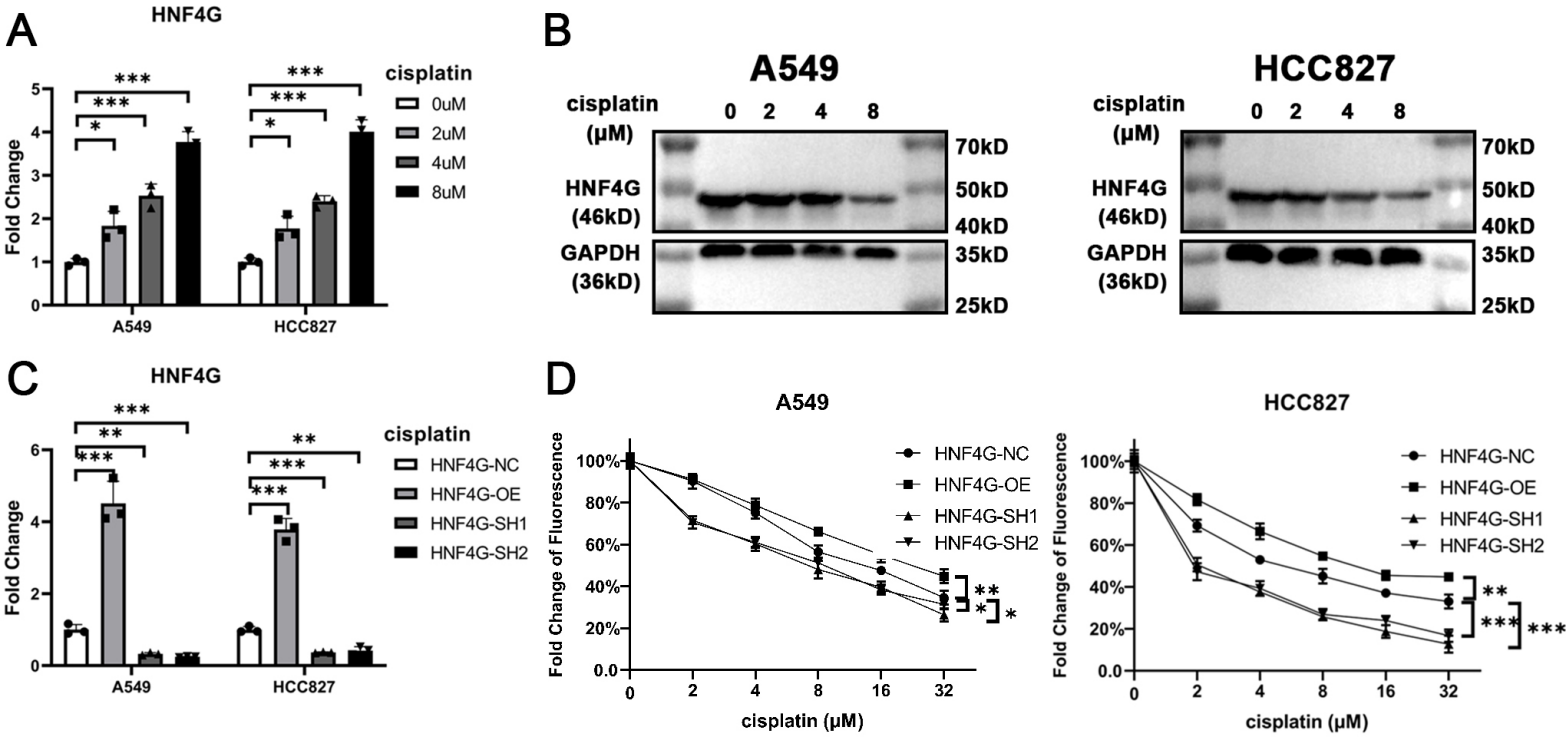
HNF4G promotes resistance to cisplatin in lung adenocarcinoma cells. (A) qRT-PCR and (B) western blot results of HNF4G expression after cisplatin treatment in A549 and HCC827 cells. (C) qRT-PCR results of HNF4G expression in HNF4G overexpression and knockdown cells. (D) Cisplatin sensitivity curves of HNF4G overexpression and knockdown cells. * *p* < 0.05, ** *p* < 0.01, *** *p* < 0.001.

We then examined the expression of HNF4G in the lung adenocarcinoma cell lines A549 and HCC827 after treatment with different concentrations of cisplatin using qRT-PCR (Fig. 2A) and western blot (Fig. 2B), and discovered that it was significantly increased after cisplatin treatment. Using lentivirus, we overexpressed and knocked down HNF4G expression in A549 and HCC827 cells, respectively (Fig. 2C). Two different shRNA sequences were used to reduce the impact of off-target effects on the experimental results. Our results showed that overexpression of HNF4G significantly enhanced the resistance of A549 and HCC827 cells to cisplatin, whereas knockdown of HNF4G had the opposite effect (Fig. 2D). According to the findings, HNF4G significantly increased the drug resistance of lung adenocarcinoma cells to cisplatin.

### HNF4G promotes MAPK6 expression and MAPK6/Akt signaling pathway

**Figure 3:**
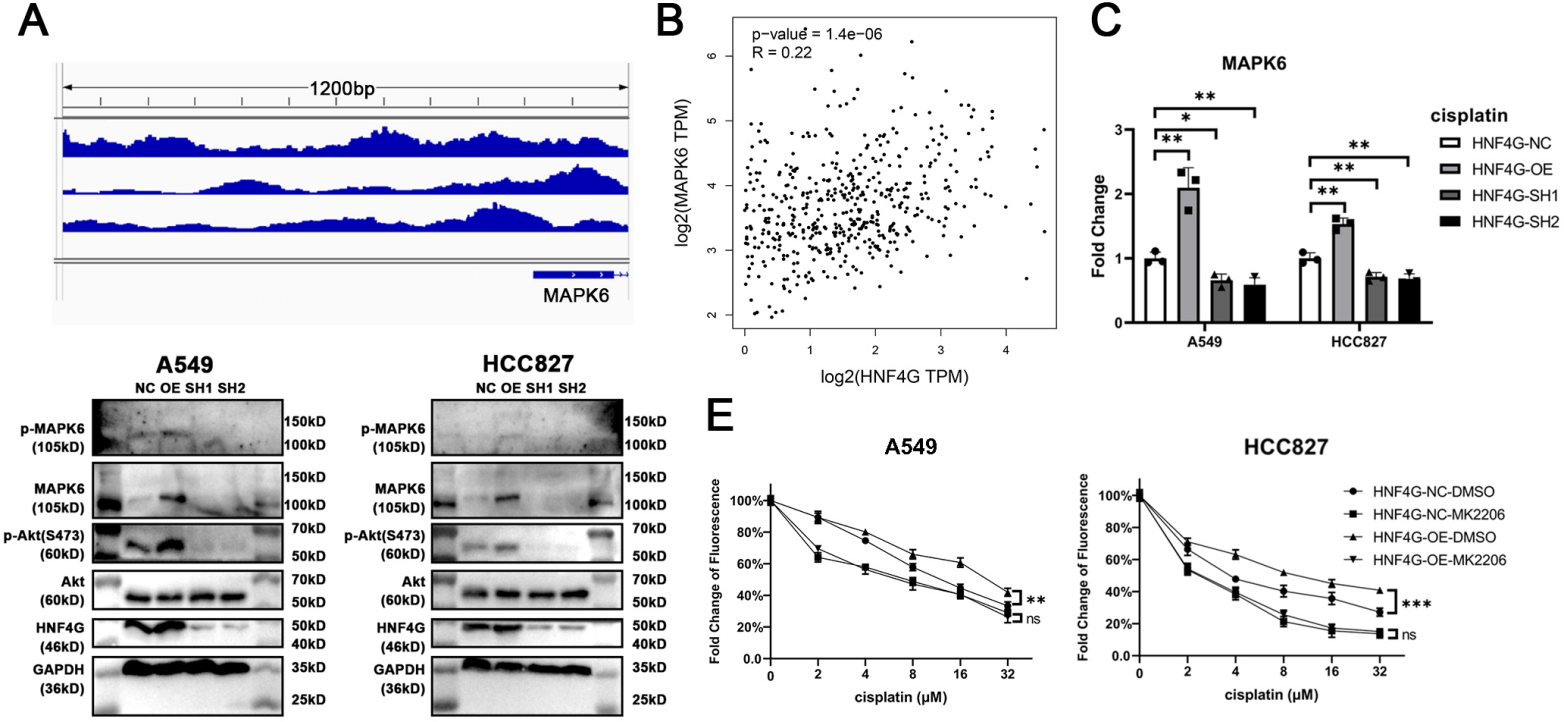
HNF4G promotes MAPK6 expression and MAPK6/Akt signaling pathway. (A) HNF4G binding peaks in the promoter of MAPK6 based on the ChIP-Seq data from the Encode database. (B) Correlation between HNF4G and MAPK6 expression in TCGA lung adenocarcinoma samples. (C) MAPK6 qRT-PCR results in HNF4G overexpression and knockdown cells. (D) Western blot analyses of HNF4G, MAPK6, p-MAPK6, Akt, and p-Akt(S473) in cells overexpressing or knocking down HNF4G. (E) Cisplatin sensitivity curves of control and HNF4G overexpression cells treated with or without Akt inhibitor.

HNF4G, as a transcription factor, may bind in the promoter regions of some cisplatin resistance genes to regulate their expression and resistance. We searched the Encode database for possible downstream factors of HNF4G in the ChIP-Seq data, looking for the target genes associated with cisplatin resistance. We discovered that there are several binding peaks of HNF4G in the −1000 bp∼200 bp promoter region upstream of the transcription start site (TSS) of MAPK6 (Fig. 3A), while previous studies have shown that MAPK6 phosphorylates the S473 site of Akt to activate Akt, and the activated significantly promotes the resistance of cells to cisplatin (Cai et al., 2021; Zhang et al., 2022; Zhou et al., 2022). Meanwhile, the RNA-Seq data from the TCGA database revealed that the expression of HNF4G was significantly positively correlated with the expression of MAPK6 in patients with lung adenocarcinoma, also indicating that HNF4G regulates the expression of MAPK6 (Fig. 3B).

Using qRT-PCR (Fig. 3C) and western blot (Fig. 3D), we confirmed that HNF4G overexpression promoted the expression and phosphorylation of MAPK6 in A549 and HCC827 cells, as well as Akt S473 phosphorylation, whereas HNF4G knockdown inhibited the expression of MAPK6 and the activation of MAPK6Akt signaling pathway.

We used 1uM MK-2206, an Akt-specific inhibitor, to inhibit the activity of the MAPK6/Akt signaling pathway. When we inhibited Akt activity, there was no longer a significant difference in cisplatin resistance between A549 and HCC827 cells overexpressing HNF4G and controls (Fig. 3E), indicating that the MAPK6/Akt signaling pathway is the key mechanism by which HNF4G acts on cisplatin resistance.

### HNF4G binds to the promoter region of MAPK6 to promote MAPK6 expression

**Figure 4:**
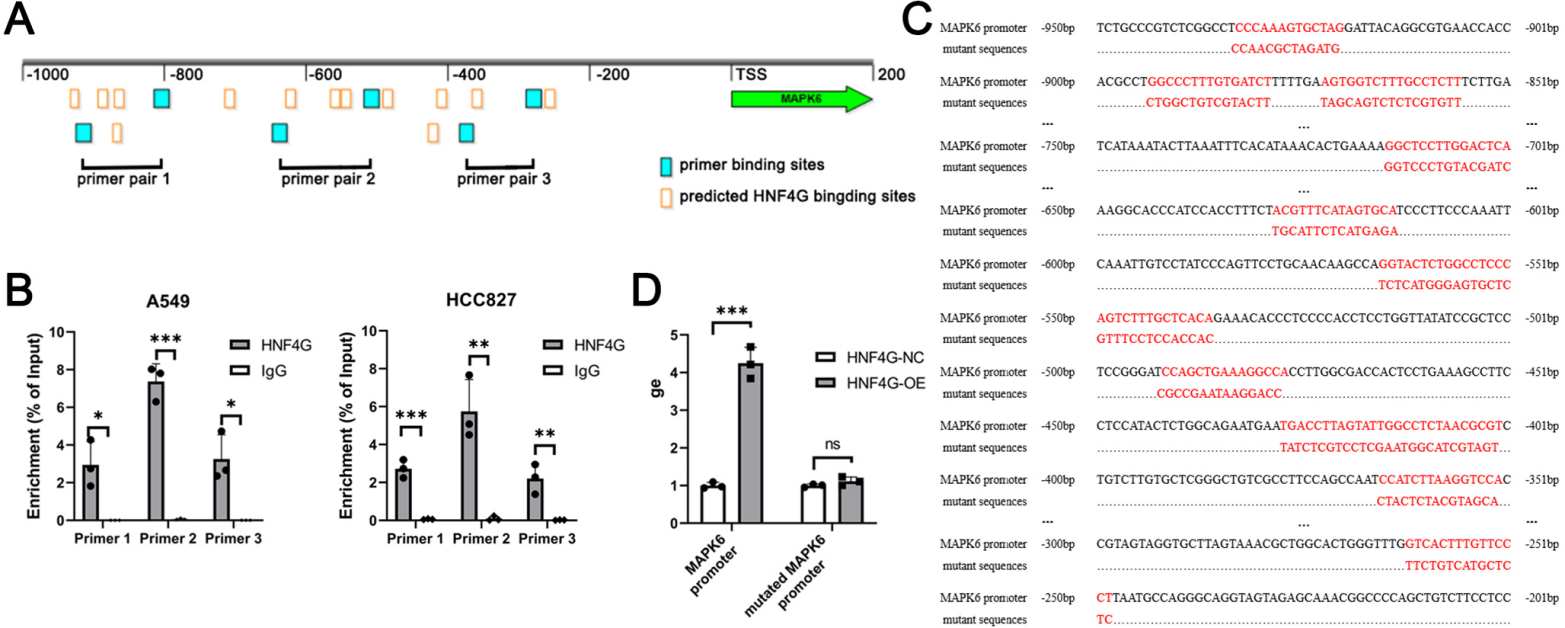
HNF4G binds to the promoter region of MAPK6 to promote MAPK6 expression. (A) Predicted HNF4G binding sites and designed primer locations in MAPK6 promoter. (B) ChIP-qPCR analyses of HNF4G’s binding in MAPK6 promoter. (C) Sequences of the potential HNF4G binding sites MAPK6 promoter and corresponding mutated sequences. (D) Luciferase assays using luciferase plasmids containing MAPK6 promoter and mutated sequences.

We obtained potential binding sites of HNF4G in the MAPK6 promoter region from the JASPAR website, and designed three pairs of primers for ChIP-qPCR to detect HNF4G binding to the MAPK6 promoter (Fig. 4A). Our ChIP-qPCR results showed that HNF4G was significantly bound to the regions around the sequences amplified by these three pairs of primers, as shown in Fig. 4B.

To conduct dual luciferase experiments, we created a firefly luciferase plasmid with the MAPK6 −1000∼200 bp region, as well as a firefly luciferase plasmid with all potential binding sites of HNF4G in this region were randomly mutated (Fig. 4C). When the plasmid contained the MAPK6 promoter, overexpression of HNF4G significantly increased the activity of the firefly luciferase (Fig. 4D). However, when all possible binding sites of HNF4G were mutated, HNF4G overexpression no longer promoted the expression of firefly luciferase (Fig. 4D).

## Discussion

The role of HNF4G in cisplatin resistance has never been reported before, nor has the regulation of MAPK6 expression by HNF4G. Based on the findings, we first discovered that HNF4G promotes MAPK6 expression and subsequent Akt phosphorylation by binding to the MAPK6 promoter region, resulting in cisplatin resistance in lung adenocarcinoma.

According to Wang et al. (2018), HNF4G expression was found to be higher in lung cancer tissues than in adjacent normal lung tissues. They also reported that HNF4G stimulated cell proliferation through the cell cycle while suppressing cell apoptosis and that Akt inhibitor could attenuate its effects (Wang et al., 2018). Our findings also confirmed that HNF4G promotes the Akt signaling pathway, and we further revealed that HNF4G activates the Akt signaling pathway in lung adenocarcinoma by increasing MAPK6 expression. Meanwhile, He et al. (2022) reported that HNF4G promoted colorectal cancer proliferation *via* the PI3K/AKT pathway by targeting GNG12 and PTK2. All of these findings indicate that the Akt signaling pathway plays an important role in HNF4G action.

HNF4G primarily promotes tumor progression, according to numerous other studies. In pancreatic cancer, HNF4G was also found to be induced by SMAD4 deficiency, promoting progression and metastasis but being suppressed by the metformin/APMK signaling pathway (Wang et al., 2021). Chen et al. (2022) discovered that HNF4G is associated with a poor prognosis in colorectal cancer and promotes tumor cell growth by inhibiting caspase-dependent apoptosis. HNF4G promotes pancreatic cancer development by increasing IGF2BP2 transcription, according to Zhan et al. (2023). By inhibiting HNF4G, miR-766-3p and miR-320b slowed tumor progression (He et al., 2022; Ma et al., 2021). Meanwhile, several studies have discovered that HNF4G collaborated with its paralog HNF4A to regulate the metabolism, differentiation, and immune function in the intestine (Chen et al., 2020; Lei et al., 2022; Girard et al., 2022; Chen et al., 2019).

Cisplatin is currently the most widely used tumor therapy drug, and it has piqued the interest of many researchers (Sui et al., 2022; Bi et al., 2022). Although newer technologies such as targeted therapy and immunotherapy are preferred in the treatment of lung adenocarcinoma, cisplatin remains useful in both mono-and combination therapies (Hu et al., 2019). One of the most important challenges to cisplatin’s application and effectiveness is resistance. A better understanding of the resistance mechanisms of cisplatin is required in order to develop more effective therapeutic options (Lv et al., 2021). We demonstrated the role of HNF4G in cisplatin resistance in lung adenocarcinoma in this study. We hope that our research will aid in promoting the effect of cisplatin therapy and improving the prognosis of patients with lung adenocarcinoma.

Our research has several limitations. First, this study only used data from chemotherapy patients in a public database and did not include our own clinical data. Second, we did not validate the effects of HNF4G on cisplatin resistance in animal models due to limited experimental conditions and funds. Third, in addition to cisplatin, several other platinum-based drugs are widely used in clinical practice, including carboplatin and oxaliplatin. Whether HNF4G increases resistance to other platinum-based drugs remains to be seen. We hope these limitations can be addressed in the future.

## Conclusions

HNF4G promotes MAPK6 expression and subsequent Akt phosphorylation by binding to the MAPK6 promoter region, resulting in resistance to cisplatin in lung adenocarcinoma.

##  Supplemental Information

10.7717/peerj.14996/supp-1 Figure S1 Raw data for Figure 1A and 1B

10.7717/peerj.14996/supp-2 Figure S2 Raw data for FIgure 2A

10.7717/peerj.14996/supp-3 Supplemental Information 3 Raw data for Figure 2C

10.7717/peerj.14996/supp-4 Supplemental Information 4 Raw data for Figure 2D

10.7717/peerj.14996/supp-5 Figure S3 Raw data for Figure 3C

10.7717/peerj.14996/supp-6 Supplemental Information 6 Raw data for Figure 3E

10.7717/peerj.14996/supp-7 Figure S4 Raw data for Figure 4B

10.7717/peerj.14996/supp-8 Supplemental Information 8 Raw data for Figure 4D

10.7717/peerj.14996/supp-9 Supplemental Information 9 Raw western blots for Figure 2B

10.7717/peerj.14996/supp-10 Supplemental Information 10 Raw western blots for Figure 3D

## Additional Information and Declarations

### Data Availability

The following information was supplied regarding data availability: The raw measurements are available in the Supplemental Files.The raw data were used in the analyses of cisplatin curves, qPCR, western blots, and luciferase reports.
