# Supplementary figures and images for: HNF4G increases cisplatin resistance in lung adenocarcinoma via the MAPK6/Akt pathway
Source: PeerJ. 2023 Mar 10;11:e14996. doi: 10.7717/peerj.14996 (PMC10010171; doi:10.7717/peerj.14996)

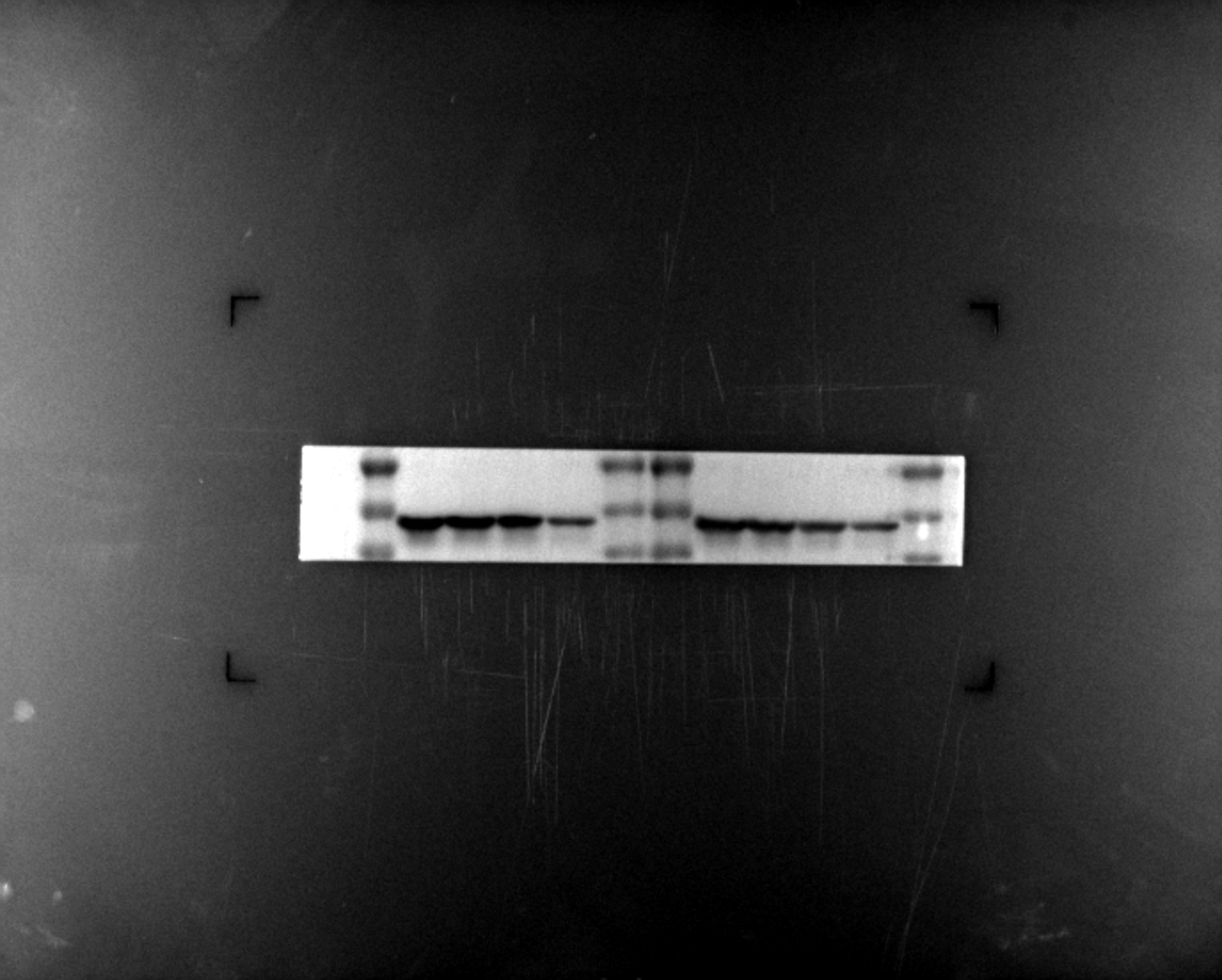

Supplement: Supplemental Information 9 [file peerj-11-14996-s009.zip › HNF4G.Tif]

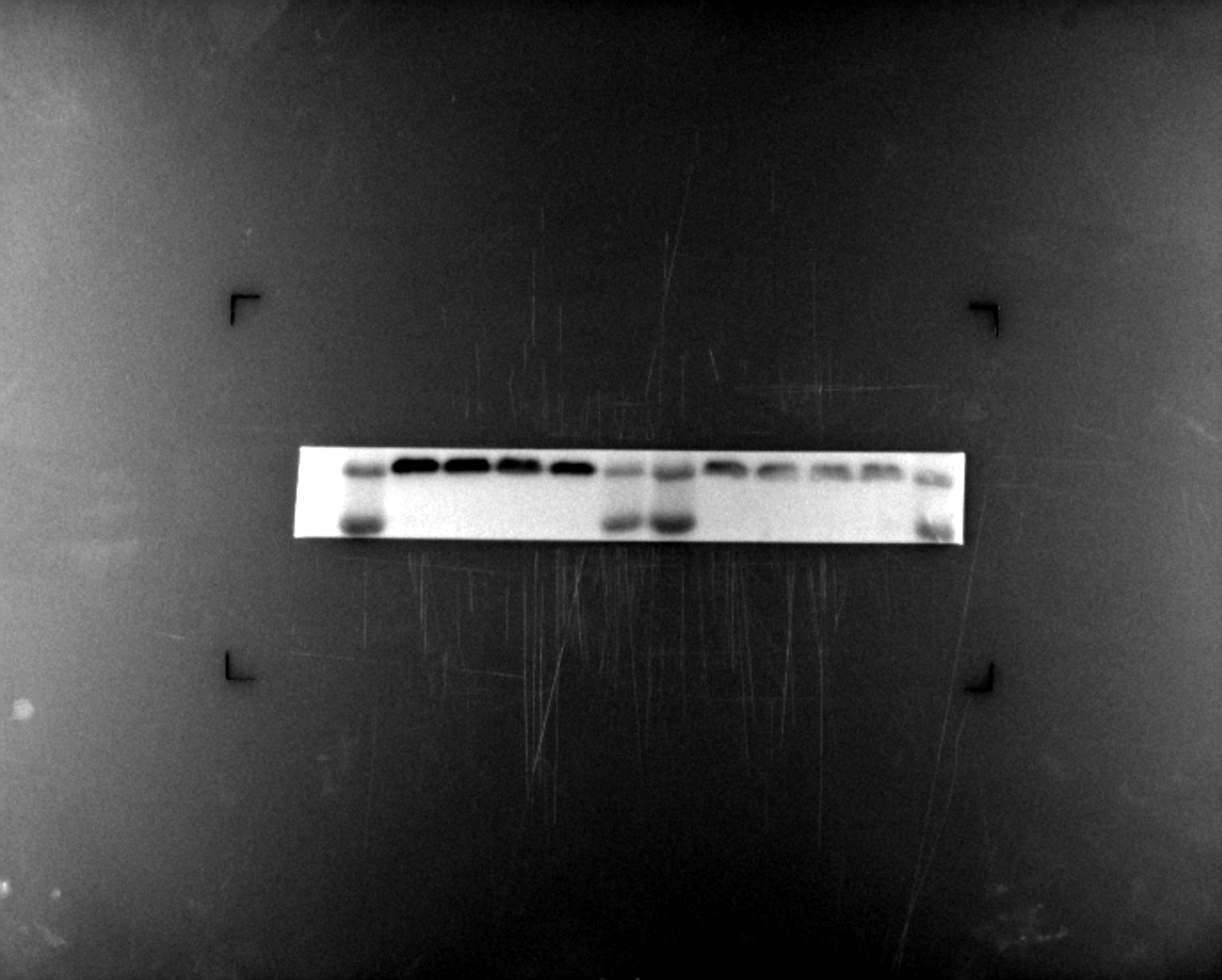

Supplement: Supplemental Information 9 [file peerj-11-14996-s009.zip › GAPDH-1.Tif]

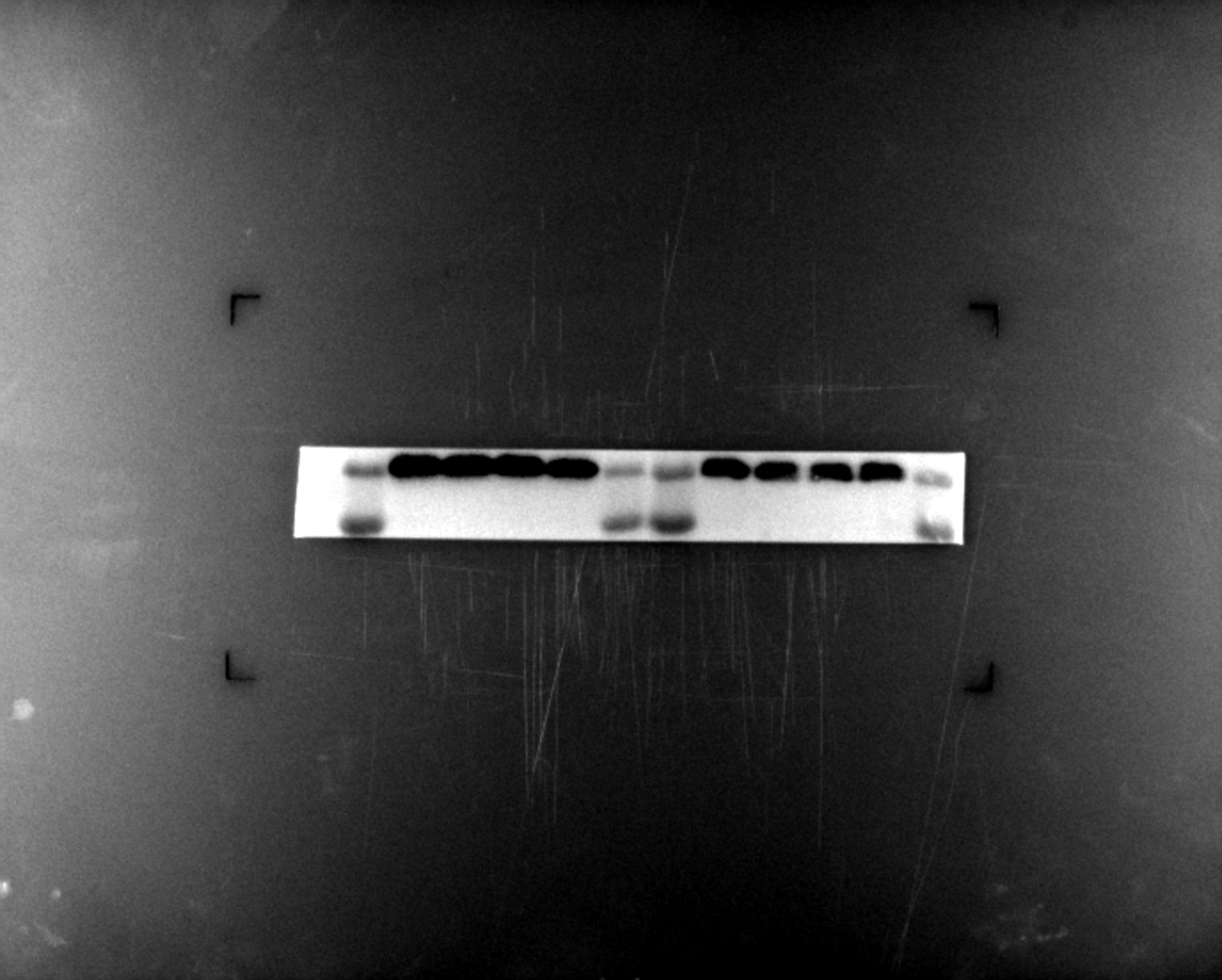

Supplement: Supplemental Information 9 [file peerj-11-14996-s009.zip › GAPDH-2.Tif]

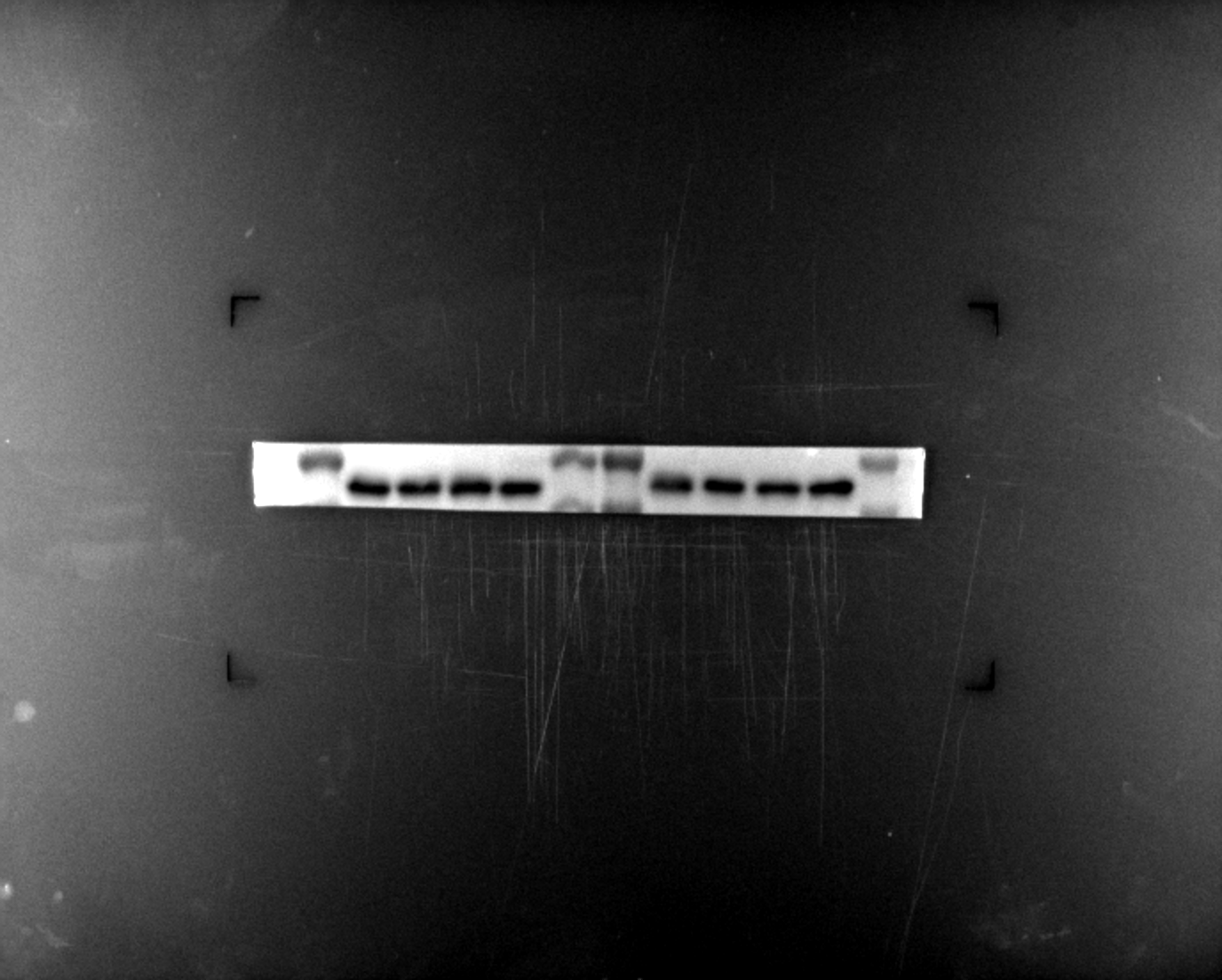

Supplement: Supplemental Information 10 [file peerj-11-14996-s010.zip › AKT.Tif]

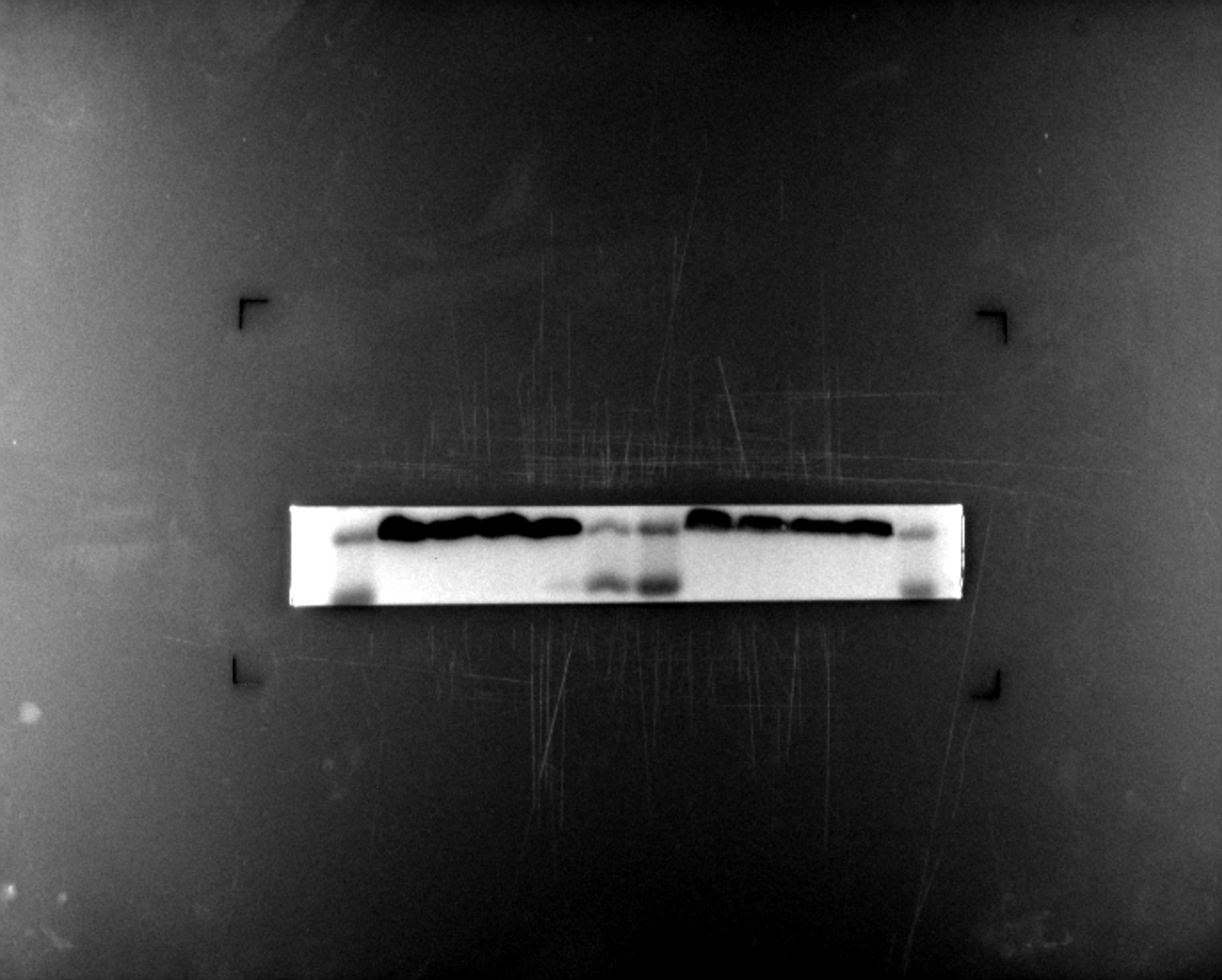

Supplement: Supplemental Information 10 [file peerj-11-14996-s010.zip › GAPDH.Tif]

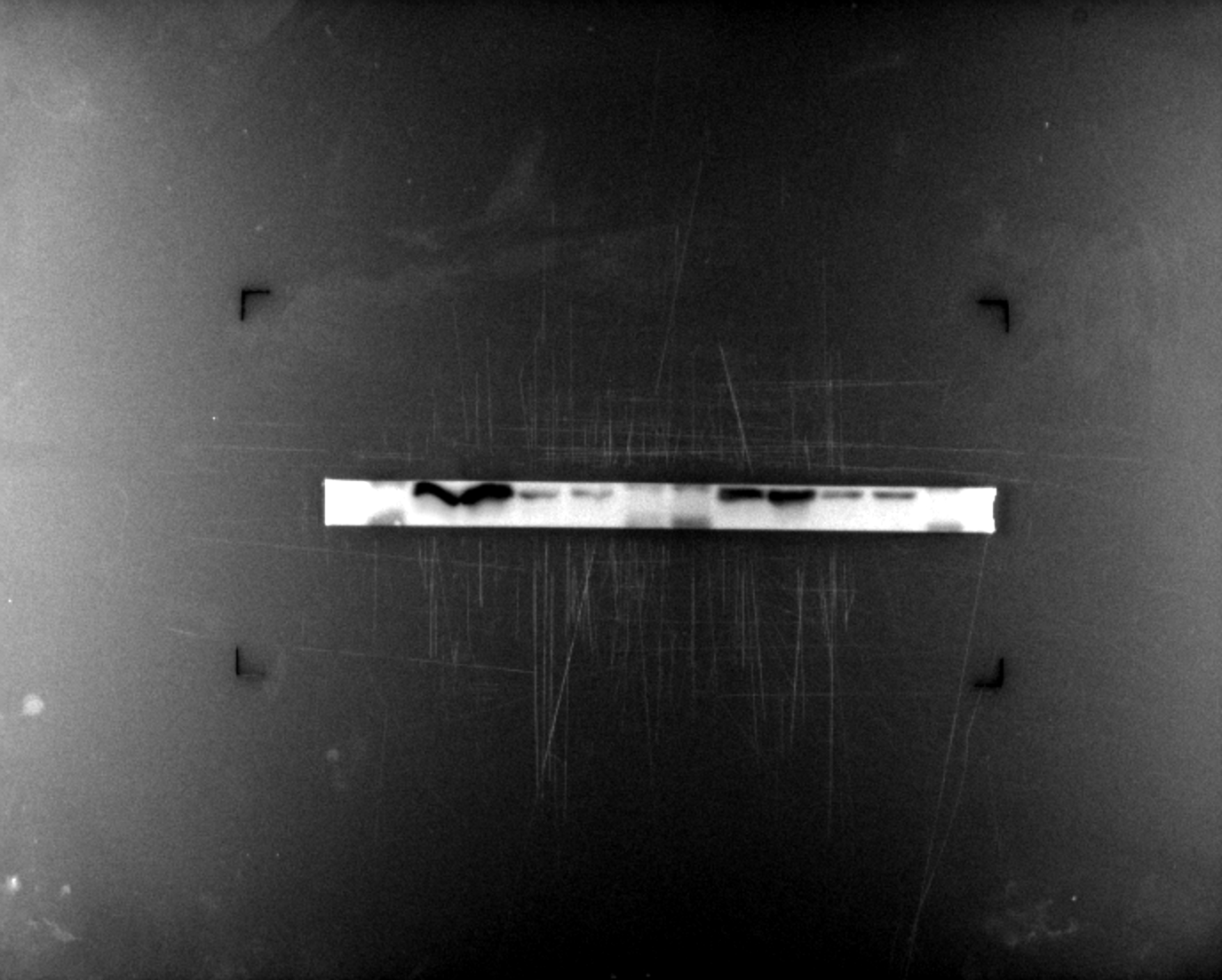

Supplement: Supplemental Information 10 [file peerj-11-14996-s010.zip › HNF4G.Tif]

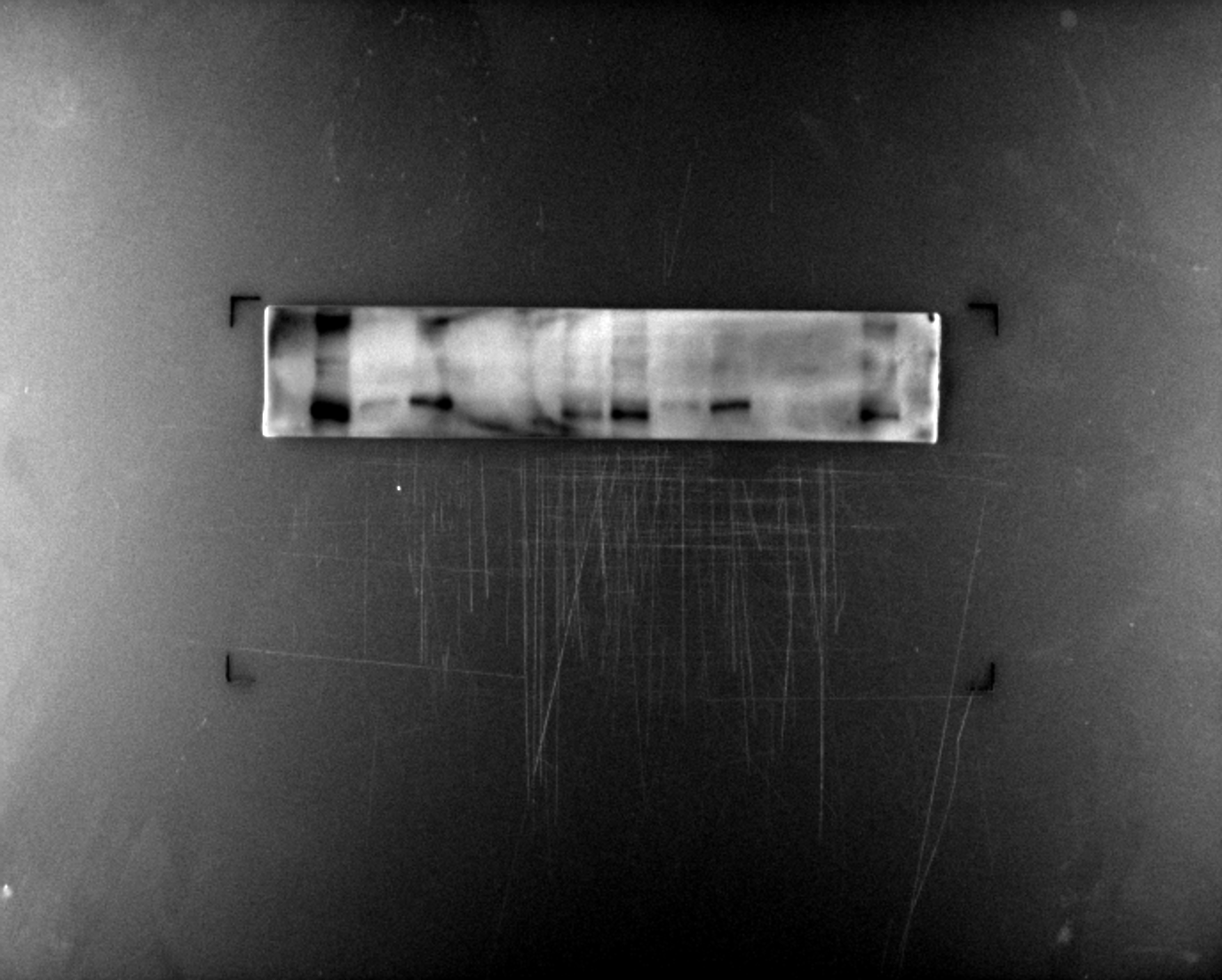

Supplement: Supplemental Information 10 [file peerj-11-14996-s010.zip › MAPK6.Tif]

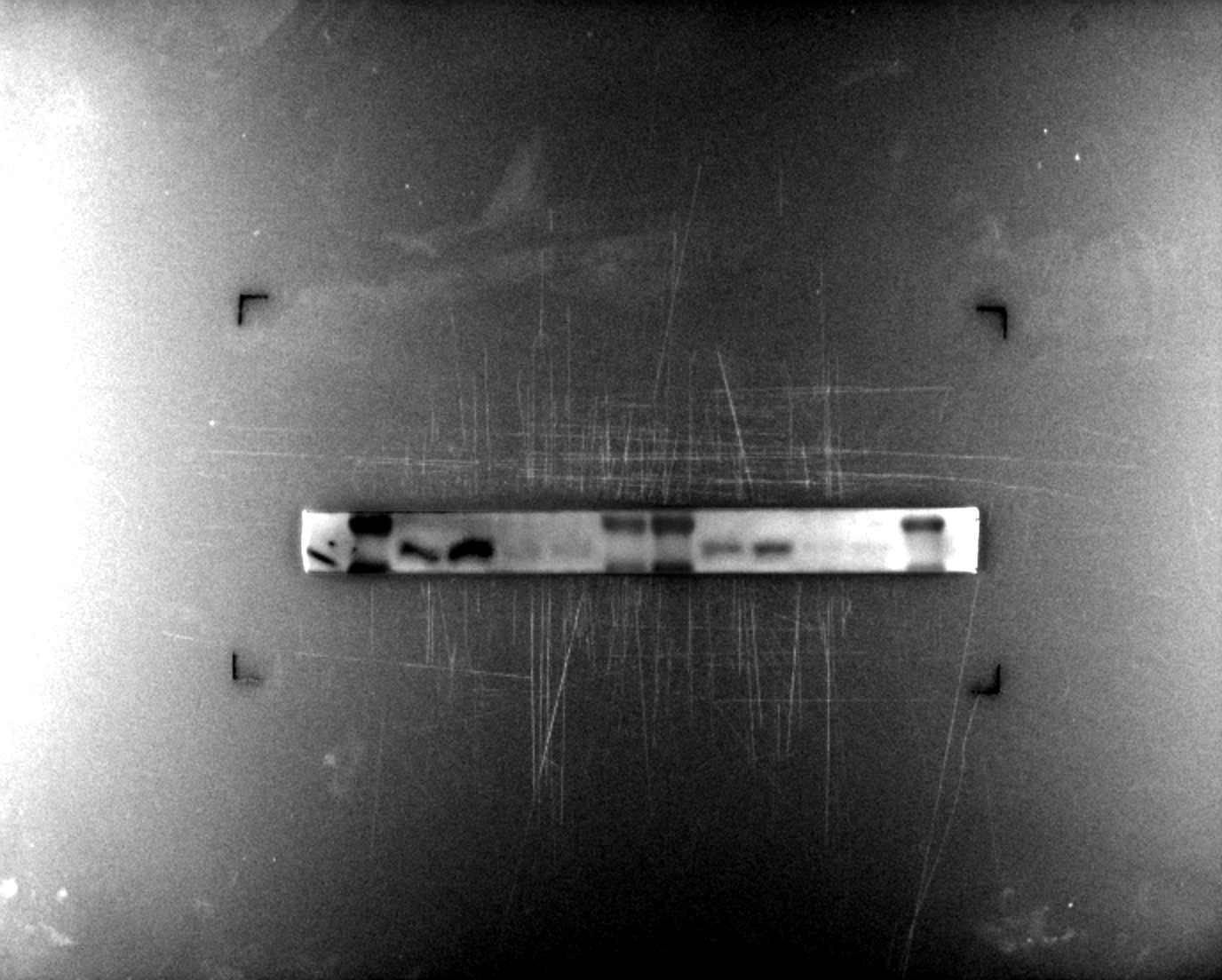

Supplement: Supplemental Information 10 [file peerj-11-14996-s010.zip › p-AKT(S473).Tif]

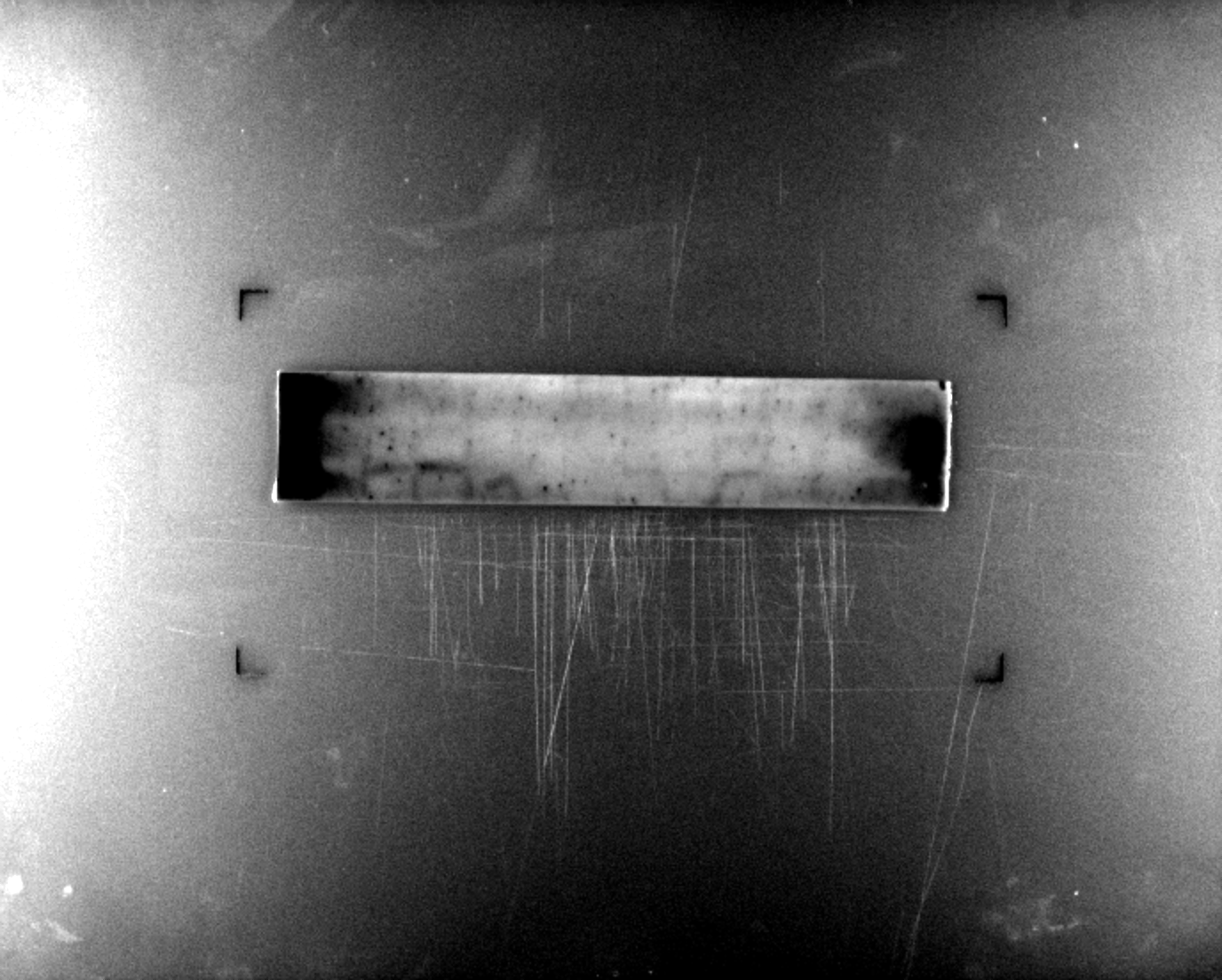

Supplement: Supplemental Information 10 [file peerj-11-14996-s010.zip › p-MAPK6.Tif]
